# Alternative Pathways for Hearing Care May Address Disparities in Access

**DOI:** 10.3389/fdgth.2021.740323

**Published:** 2021-11-25

**Authors:** Amber Willink, Lama Assi, Carrie Nieman, Catherine McMahon, Frank R. Lin, Nicholas S. Reed

**Affiliations:** ^1^Menzies Centre for Health Policy, University of Sydney, Sydney, NSW, Australia; ^2^Cochlear Center for Hearing and Public Health, Johns Hopkins University Bloomberg School of Public Health, Baltimore, MD, United States; ^3^Department of Otolaryngology, Johns Hopkins School of Medicine, Baltimore, MD, United States; ^4^Department of Linguistics, Macquarie University, Sydney, NSW, Australia; ^5^Department of Epidemiology, Johns Hopkins University Bloomberg School of Public Health, Baltimore, MD, United States

**Keywords:** hearing impairment, hearing aid, direct to consumer, health services utilization, older adults

## Abstract

**Background/Objectives:** Low-uptake of hearing aids among older adults has long dogged the hearing care system in the U.S. and other countries. The introduction of over-the-counter hearing aids is set to disrupt the predominantly high-cost, specialty clinic-based delivery model of hearing care with the hope of increasing accessibility and affordability of hearing care. However, the current model of hearing care delivery may not be reaching everyone with hearing loss who have yet to use hearing aids. In this study, we examine the group of people who do not use hearing aids and describe their characteristics and health care utilization patterns. We also consider what other healthcare pathways may be utilized to increase access to hearing treatment.

**Design:** Cross-sectional, the 2017 Medicare Current Beneficiary Survey.

**Setting:** Non-institutionalized adults enrolled in Medicare, the U.S. public health insurance program for older adults (65 years and older) and those with qualifying medical conditions and disabilities.

**Participants:** A nationally representative sample of 7,361 Medicare beneficiaries with self-reported trouble hearing and/or hearing aid use.

**Measurements:** Survey-weighted proportions described the population characteristics and health care utilization of those with hearing loss by hearing aid use, and the characteristics of those with untreated hearing loss by health care service type utilized.

**Results:** Women, racial/ethnic minorities, and low-income Medicare beneficiaries with self-reported hearing trouble were less likely to report using hearing aids than their peers. Among those who do not use hearing aids, the most commonly used health care services were obtaining prescription drugs (64%) and seeing a medical provider (50%). Only 20% did not access either service in the past year. These individuals were more likely to be young and to have higher educational attainment and income.

**Conclusion:** Alternative models of care delivered through pharmacies and general medical practices may facilitate access to currently underserved populations as they are particularly high touch-points for Medicare beneficiaries with untreated hearing trouble. As care needs will vary across a spectrum of hearing loss, alternative models of hearing care should look to complement not substitute for existing access pathways to hearing care.

## Impact Statement

We certify that this work is novel and provides important contributions to the literature by highlighting the disparities in access to hearing care and alternative pathways of providing hearing care that could address existing disparities among Medicare beneficiaries.

## Key Points

- Women, racial/ethnic minorities, and low-income Medicare beneficiaries are less likely to use hearing aids.
- They more commonly visit pharmacies and general medical practices for health care.
  Why does this matter?
  Less that one in five Medicare beneficiaries with hearing loss use hearing aids. Delivering hearing care through alternative pathways may improve hearing care access.

## Introduction

In 2020, 44.11 million adults in the U.S. were estimated to have hearing loss; with the aging of the population, the number is expected to increase to 73.50 millions in the next 40 years (1). Hearing loss, the enact of encoding peripheral environmental auditory information for central decoding in the brain, can have a great toll on well-being and communication (2), and may lead to poor quality of life (3), and disability (4). It has also been associated with negative health consequences such as increased risk of falls (5), and cognitive decline (6), and has been recognized as a modifiable risk factor for dementia (7).

Hearing aids have been shown to improve hearing-related quality of life, listening ability, communication, and social and emotional function (8, 9). Early observational studies suggest that hearing aids may improve cognitive functions by preventing auditory deprivation which can result in insufficient cognitive stimulation (10, 11). Yet, despite the association of hearing loss with negative health consequences, and potential benefits of hearing aid use, <15% of adults with hearing loss aged 50 and over in the U.S. report using hearing aids (12).

Barriers to hearing aid use include high cost and lack of, or inadequate, insurance coverage, perceived stigma by others, significant resources required to navigate current processes, including transportation, mobility, and know-how as well as a lack of clear recommendations or guidance by primary care providers (13, 14).

Current hearing care delivery in the U.S. follows a medical model of specialty clinic-based care, primarily through audiologists and otolaryngologists, which may be costly and time-consuming; even those who have the time and money needed may find it frustrating (15). The current model is grounded in dispensing hearing aids through licensed individuals which requires multiple visits to a hearing aid dispenser or audiologist for identification of hearing needs, customization of the product, and continual maintenance and fine-tuning. It is also common for the services of the professional to be bundled into the sale of the hearing aid as a markup. When this hearing delivery model was developed in the 1970's, hearing aids were extremely complicated and potentially produced dangerous noise levels. This required in-person fitting and tuning by trained professionals. Advances in technology have increasingly allowed for integration and easy adjustment via smartphone, opening the possibilities for alternative models of care.

Alternative approaches to the specialty clinic-based hearing care model tackle some of the shortcomings of the typical clinic-based model, and include community-delivered hearing care, mobile health applications, tele-audiology, pharmacies, retail clinics such as Costco and Walgreens, and involve primary care providers in hearing care (16). Community-delivered hearing care models can include community health workers, peer educators, community health aides, among other trained paraprofessionals who can provide education on hearing loss, basic aural rehabilitation, as well as fitting and orientation to OTC devices (17–19). Some large retail clinics in the United States, such as Costco and Sam's Club, have integrated hearing aid centers into their stores. This model generally recreates best-practice hearing aid delivery models used in private clinics but increases accessibility by putting the clinic where customers already are shopping and increases affordability by leveraging buying power from the large corporations.

Recognizing the importance of treating hearing loss and the presence of barriers to hearing care access at the national level, the President's Council of Advisors on Science and Technology and the National Academies of Sciences, Engineering, and Medicine released reports with recommendations to improve the accessibility of hearing care for older adults (16, 20), and in 2017, the Over-the-Counter (OTC) Hearing Aid Act law was passed with the aim of making hearing aids more accessible and affordable to those with mild or moderate hearing loss (21).

The OTC Hearing Aid Act required the Food and Drug Administration (FDA) to develop regulations by August 2020 for the sale of over-the-counter hearing aids to treat mild to moderate hearing loss. The FDA has missed this statutory deadline but are scheduled to release these recommendations by the end of the year (22).

By allowing the sale of OTC hearing aids that would be regulated to ensure the safety and efficacy of these devices, people with self-perceived hearing loss will be able to purchase hearing aids without assessment or counseling from a hearing care professional. However, ensuring proper access to hearing care services is essential to promote optimal hearing loss management and maximal benefit from hearing aids for those who need them. In a randomized-controlled trial, people who self-selected their own pre-programmed hearing aids via an OTC service-delivery model, compared to those who received hearing aids via an audiologist-based service-delivery model, were less likely to be satisfied with their hearing aid or purchase one after the study (23).

From a public health perspective, it is unclear to what extent alternative models currently available reach people with hearing loss who have yet to use hearing aids. People with untreated hearing loss have different health care utilization patterns and costs compared to those without hearing loss, (24) and compared to those with hearing loss who use hearing aids (25). Those with untreated hearing loss are more likely to visit emergency departments (24), report unmet health care needs (26), and not have a usual source of care (14). Understanding how people with untreated hearing loss access the general health care system and what characteristics set them apart from those who already use hearing aids is fundamental to understanding how we can reach those Medicare beneficiaries with hearing loss who are not served by the current model of care.

In this study, we identify who the current model of hearing care is serving and how those individuals differ from Medicare beneficiaries with untreated hearing trouble. We then explore the other health care service patterns of the population with untreated hearing trouble and describe the populations accessing the most common health care services by sociodemographic characteristics and health status, to demonstrate the population that could be potentially reached via alternative delivery models of hearing care. We focus in on medical providers and pharmacies as those services have the highest utilization across the Medicare population.

## Methods

### Study Sample

We used the 2017 Medicare Current Beneficiary Survey and Cost Supplement file, a nationally representative survey of Medicare beneficiaries linked to administrative claims data. Medicare is the publicly-funded health insurance program in the United States for adults aged 65 years and older and those under 65 years who qualify based on medical condition or disability. For this study, 7,361 Medicare beneficiaries who self-reported a little or a lot of trouble hearing or reported using hearing aids were included in the analytic sample.

### Measures

The analysis was separated into two sub-populations: untreated functional hearing impairment (those who self-reported a little or a lot of trouble hearing but no hearing aid use), and those who did report using a hearing aid.

The primary outcome of interest was healthcare utilization by service type, including inpatient, outpatient (e.g., hospital outpatient department or clinic visits), medical provider (e.g., physician, primary care, or allied health), prescription drugs, home health, and skilled nursing facilities services. These utilization variables were derived from both survey report and administrative claims data and through an adjudication process developed by the MCBS administrators (27). As the objective of this analysis is to assess in-person utilization of services as possible avenues for hearing care treatment, we have refined the measure of medical provider to be limited to those most likely seen in a primary care setting, including medical doctors (excluding specialists), nurse practitioners, and physicians' assistants. This measure was refined as delivering hearing care through a primary care service, rather than through specialists, is more practical and within the scope of general practice. We have also refined the prescription drug category to identify those who visit a pharmacy to receive their prescriptions. If a Medicare beneficiary reported often receiving their prescriptions in the mail or *via* the internet, they were counted as not having a prescription drug touch point as we are trying to identify in-person visits that might lend themselves to receiving other healthcare services.

Medicare beneficiaries who reported untreated functional hearing loss were described according to the health care service types utilized. Population characteristics used to describe the groups included sociodemographic characteristics, health and functional status, and access to online information. Sociodemographic characteristics included age, gender, race/ethnicity, educational attainment, income relative to federal poverty level, living arrangement, urban/rural status, and supplemental insurance coverage. Supplemental insurance coverage includes Medicaid for low-income adults, Medicare Advantage (the private arm of Medicare), and Medigap the supplemental plans that correspond with the public program. Medicare Advantage plans may include coverage of hearing aids. Health-related variables included severity of hearing trouble, functional vision impairment, number of chronic conditions, cognitive impairment, number of activities of daily living limitations (ADLS), and having a helper to carry out ADLs. Access to online information included having a personal computer at home and using the Internet to get information.

### Statistical Analysis

This study is a descriptive analysis of socio-demographic characteristics among Medicare beneficiaries with untreated hearing loss across different groups based on health care utilization. All analyses used the survey weights provided by the Centers for Medicare and Medicaid to account for the complex survey design of the MCBS including the over-sampling of black and Hispanic populations, and survey non-response. Weighted proportions were used to describe the characteristics and health care service utilization of Medicare beneficiaries with functional hearing loss by hearing aid use, and the characteristics of people with untreated functional hearing loss by health care service type utilized. Group comparisons were made using Pearson's chi-squared test of independence. StataSE 14 was used to conduct all analyses.

## Results

### Descriptive Characteristics

Overall, 7,361 Medicare beneficiaries reported hearing trouble or hearing aid use, representing a weighted sample of 28,195,657 Medicare beneficiaries. Among them, 87% reported not using hearing aids (Table 1). A greater proportion of persons who did not use hearing aids compared to those who did were young, women, and non-White. Those who did not use hearing aids were also more likely to have lower educational attainment and lower income. A greater proportion of those not using hearing aids had two or more limitations in activities of daily living compared to those using hearing aids.

**Table 1:** Characteristics of Medicare beneficiaries with functional hearing trouble by hearing aid use.

	**Untreated functional hearing trouble**	**Hearing trouble with hearing aid**
Unweighted sample (*n*)	5,139	2,222
Weighted population (*N*)	20,803,386	7,392,271
Population distribution (%)	87%	13%
Age	Column percentages	
<65	14%	4%
65-74	48%	37%
75-84	26%	33%
85+	11%	26%
Women	51%	40%
**Race/ethnicity**		
White	86%	92%
Black	9%	4%
Hispanic	2%	1%
Asian	2%	1%
Other	2%	2%
**Education**		
Less than HS	16%	12%
HS Graduate	52%	50%
Completed College	32%	38%
**Income relative to FPL**		
<100%	14%	7%
100-149%	14%	10%
150-199%	11%	10%
200-399%	28%	32%
400%+	32%	41%
**Number of chronic conditions**		
0	7%	5%
1-2	36%	35%
3-5	44%	47%
6+	13%	12%
**Number of ADLs**		
0 ADLs	65%	70%
1 ADL	15%	14%
2+ ADLs	20%	16%

*ADLs, activities of daily living; FPL, federal poverty level; HS, high school. Source: Authors' analysis of the Medicare Current Beneficiary Survey 2017. All characteristics have statistically significant distributions between those with hearing aids and those without at p < 0.05, column percentages may not add up to 100% due to rounding*.

### Health Care Utilization by Service Type

**Figure 1:**
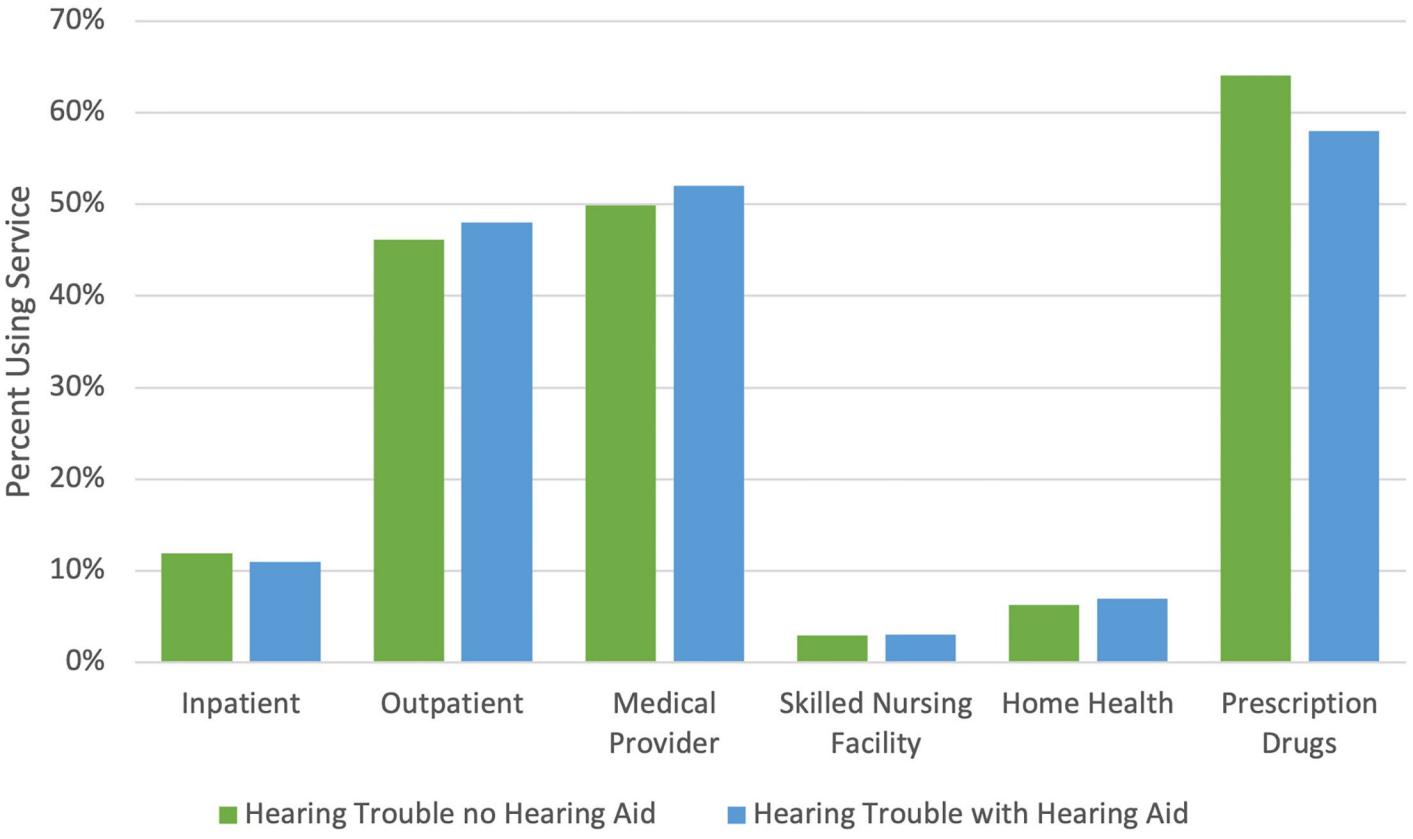
Percent of Medicare beneficiaries with functional hearing trouble who report any utilization in 2017 by service type and hearing aid use. Source: Authors' analysis of the Medicare Current Beneficiary Survey 2017. Medical provider includes medical doctor, nurse practitioner, physician assistant, and nurses. Prescription drugs do not include beneficiaries who report often receiving their prescription drugs in the mail or through the internet.

The health care services most accessed by Medicare beneficiaries with hearing trouble in 2017 were obtaining prescription drugs, seeing a medical provider, and utilizing outpatient services (Figure 1). Among those with untreated functional hearing loss, 64% reported obtaining prescription drugs, 50% saw a provider, and 46% accessed outpatient services. Less commonly, they accessed inpatient services (12%), home health (6%), and skilled nursing facilities (3%). The distribution of health care utilization by service type was similar among those who used hearing aids.

**Figure 2:**
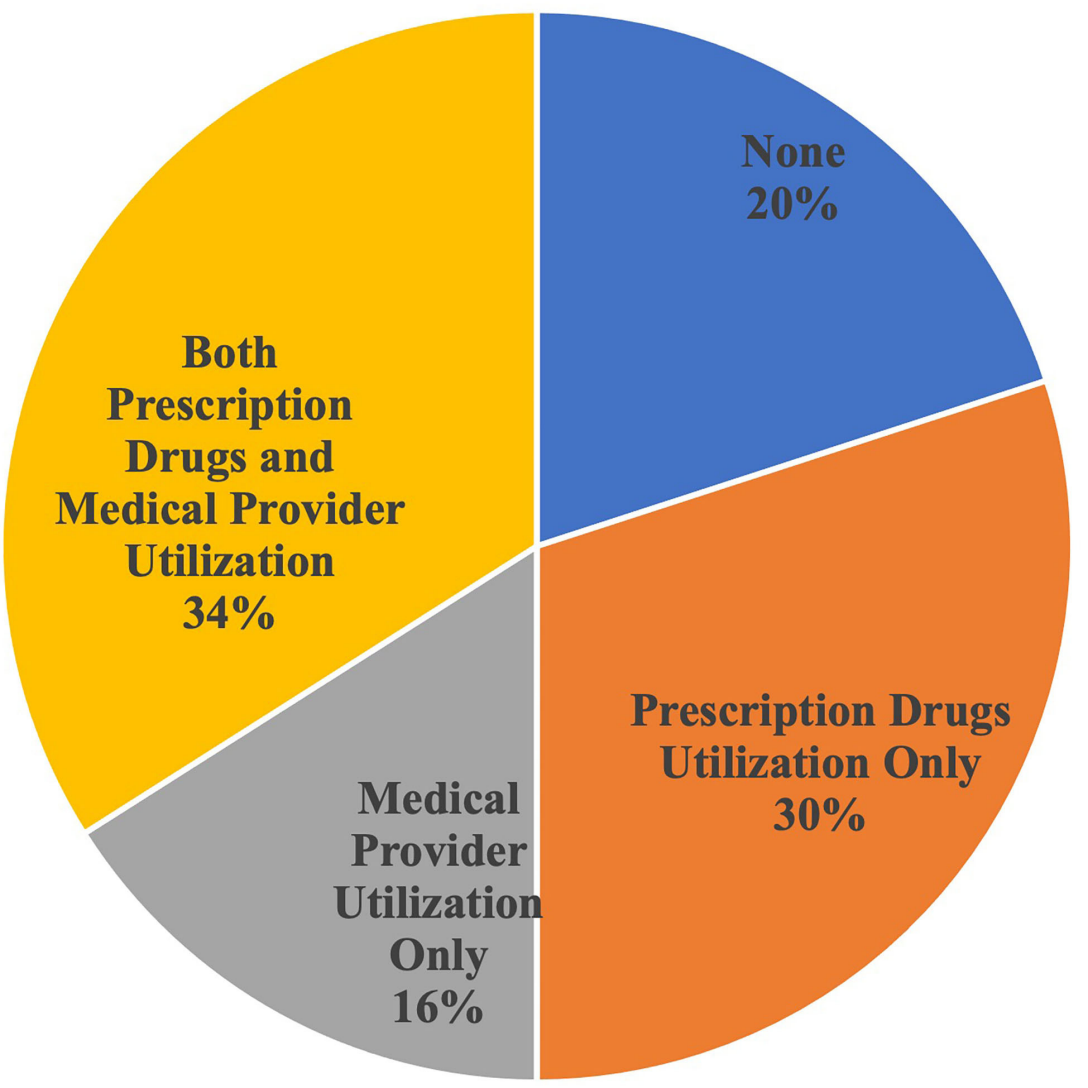
Intersection of medical provider and prescription drug utilization by Medicare beneficiaries with untreated functional hearing trouble. Source: Authors' analysis of the Medicare Current Beneficiary Survey 2017. Medical provider includes medical doctor, nurse practitioner, physician assistant, and nurses. Prescription drugs do not include beneficiaries who report often receiving their prescription drugs in the mail or through the internet.

Figure 2 shows the extent of the utilization overlap among the two most common services types. Among the Medicare beneficiaries with untreated functional hearing loss, 34% saw a medical provider and obtained prescription drugs from a pharmacy in the past year, 16% saw a medical provider only, and 30% only obtained prescription drugs from a pharmacy. Twenty percent did not access any of these two health care service types.

### Characteristics of Medicare Beneficiaries With Untreated Functional Hearing Loss by Health Care Services Utilized

The characteristics of Medicare beneficiaries with untreated functional hearing loss are presented by service type utilized (Table 2). Fifty percent of Medicare beneficiaries with untreated functional hearing loss saw a medical provider in the past year. Compared to those who did not see a medical provider, those who did had a greater proportion of individuals who were older, women, living alone, and with supplemental insurance coverage, especially Medicare Advantage. They also had a greater proportion with a high number of chronic conditions, functional vision impairment, and cognitive impairment, but fewer reporting limitations in activities of daily living or to have a helper. A larger proportion of those who did see a provider, compared to those who did not, had a personal computer at home, but a similar proportion in both groups used the Internet to access information.

**Table 2:** Characteristics of Medicare beneficiaries with untreated functional hearing trouble by service utilization, 2017.

	**Any medical provider or prescriptive drug use**		**Medical provider visit**		**Prescriptive drugs**	
	**None**	**Either MP or PD Use**	**No**	**Yes**	**No**	**Yes**
Unweighted sample	873	4,266	2,423	2,680	1,636	3,467
Weighted population	4,214,409	16,587,213	10,432,013	10,369,609	7,480,263	13,321,359
Population distribution (%)	20%	80%	50%	50%	36%	64%
**Age**						
<65	12%	15%***	15.38%	13%***	11%	16%***
65-74	58%	45%	49%	48%	55%	44%
75-84	22%	27%	26%	27%	24%	28%
85+	8%	12%	10%	13%	10%	12%
Women	40%	54%***	49%	53%***	42%	56%***
**Race/ethnicity**						
White	88%	83%*	84%	85%	89%	82%***
Black	7%	9%	8%	8%	6%	10%
Other	5%	8%	8%	7%	5%	9%
**Education**						
Less than high school	12%	17%***	17%	15%	10%	19%***
High school graduate	53%	52%	53%	52%	52%	53%
Completed college	35%	31%	31%	33%	38%	28%
**Income relative to FPL**						
<100%	9%	15%***	14%	13%	7%	18%***
100-149%	11%	15%	15%	14%	10%	17%
150-199%	10%	12%	11%	11%	10%	12%
200-399%	28%	28%	27%	29%	29%	28%
400%+	43%	30%	33%	32%	45%	25%
**Living arrangement**						
Alone	23%	28%***	26%	28%***	24%	28%***
Spouse	57%	49%	50%	51%	58%	46%
Children/family	11%	15%	14%	14%	10%	17%
Other	9%	8%	10%	7%	8%	9%
Rural	25%	23%	23%	23%	24%	23%
Cognitive impairment	6%	11%***	8%	12%***	8%	10%***
**Supplemental insurance coverage**						
Medicare only	31%	11%***	18%	13%***	28%	8%***
Medicaid	5%	21%	18%	17%	4%	25%
Employer	36%	17%	21%	20%	37%	12%
MA	14%	28%	23%	28%	15%	31%
Medigap	14%	23%	19%	22%	16%	24%
**Has trouble hearing**						
A little trouble	91%	88%*	89%	88%	91%	88%***
A lot of trouble	9%	12%	11%	12%	9%	12%
Has trouble with vision	37%	47%***	44%	47%*	40%	48%***
**Number of chronic conditions**						
0	13%	5%	9%	5%***	11%	5%***
1-2	44%	34%	40%	31%	40%	33%
3-5	35%	47%	40%	49%	40%	47%
6+	8%	14%	11%	15%	9%	15%
**Number of ADLs**						
0 ADLs	71%	64%***	64%	67%*	71%	62%***
1 ADL	14%	15%	15%	14%	15%	15%
2+ ADLs	15%	21%	21%	19%	14%	23%
Has a helper	28%	34%***	34%	32%*	27%	36%***
Has a personal computer at home	76%	67%***	67%	70%*	78%	64%***
Ever use the Internet to get info	70%	61%***	62%	63%	70%	58%***

ADLs, activities of daily living; FPL, federal poverty level; HS, high school; MP, medical provider; PD, prescription drugs. Source: Authors' analysis of the Medicare Current Beneficiary Survey 2017. P-value < 0.001*** < 0.01** < 0.05*.

Sixty-four percent of Medicare beneficiaries with untreated functional hearing loss obtained prescription drugs in the past year. Compared to those who did not, a greater proportion of those who obtained prescription drugs were non-White, a woman, and younger than 65 years old and older than 75 years. A larger proportion of individuals had low educational attainment and income, had supplemental insurance coverage, especially Medicaid, Medicare Advantage, or Medigap, were living alone or with children and other family members, rather than living with a spouse. A larger proportion of people who obtained prescription drugs in the past year relative to those who did not obtain prescription drugs had chronic conditions, cognitive impairment, a lot of trouble (rather than a little trouble) hearing, trouble seeing, limitations in activities of daily living, and had a helper. Compared to those who did not obtain prescription drugs, fewer Medicare beneficiaries who did obtain prescription drugs reported having a computer or using the internet for information.

Among Medicare beneficiaries with untreated functional hearing loss, 20% did not see a medical provider or obtain prescription medicines in the past year. Compared to those who utilized any of these services, those who did not were younger (<65 years old), White, men and had higher educational attainment and income. Among those who did not obtain prescription drugs or see a medical provider in the past year, 69% did not have supplemental insurance coverage, compared to 89% of those who utilized at least one of the services, and 57% lived with a spouse, compared to 49%. A smaller proportion of those who did not access any of the services compared to those who did had chronic conditions (87 vs. 95%), cognitive impairment (6 vs. 11%), limitations in activities of daily living, a lot of trouble hearing (rather than a little trouble hearing), trouble with vision, and had a helper. Of those who did not obtain prescription drugs or see a medical provider in the past year, 76% had a personal computer at home and 70% used the Internet to get information, compared to 67 and 61%, respectively, among those who accessed any of the services.

## Discussion

This study reinforces the low uptake of hearing aids among Medicare beneficiaries who report having hearing trouble and the differences in socio-demographic characteristics and health services utilization between those who use hearing aids and those who do not. Almost nine in ten (87%) beneficiaries who identify hearing problems are not serviced by the current model of hearing care, which involves high-cost devices and a predominantly specialty clinic-based approach to hearing care. Those with untreated functional hearing loss are more often women and racial/ethnic minorities. They have lower incomes, and more functional limitations than those who use hearing aids. This corroborates the financial and physical barriers to accessing hearing aids as found in previous analyses (13, 28).

Many studies highlight the importance of hearing to one's health and well-being. From a health system perspective, untreated hearing loss is associated with higher health care costs and poor health outcomes (24). The current model of care is not serving the majority of those in need. In fact, among minority groups access to hearing treatment has decreased over the last decade (29). Alternative pathways need to be considered. Some alternative delivery models are currently being piloted in the community [e.g., HEARS (17), Oyendo Bien (19)], through retail clinics [e.g., Walgreens, COSTCO (30)], and within adult day clinics [e.g., PACE clinics (31)] and tele-health (32–34) [e.g., Veterans Affairs (35)] (see Supplementary Table 1 for more detail).

While these alternative delivery models are being trialed in specific locations, this analysis provides a nationally representative picture of health care access patterns among Medicare beneficiaries with self-reported hearing trouble to provide insights into which alternative models of hearing care may be the most accessible to these individuals. Sixty-four percent of beneficiaries with untreated functional hearing trouble visited a pharmacy and 50% visited a medical provider. Our analysis suggests that a hearing care program run through a pharmacy would reach a population with greater financial and physical barriers, than a program run through a medical provider clinical only. Those who visited the pharmacy were more likely to be lower income, in a minority racial or ethnic group, with greater comorbidities, and functional limitations, than those who did not go to the pharmacy. Pharmacies have taken on a greater role in delivering health care to communities over time across other aspects of health including administering vaccinations, medication reconciliation, and patient education (36).

Interestingly, 20% of the untreated functional hearing trouble group did not visit a pharmacy or medical provider in the previous 12 months, suggesting that these approaches would not capture all beneficiaries with self-reported hearing trouble. These non-users tended to be aged 65-74, men, white, with higher income and living with a spouse. They were more likely to report a little rather than a lot of hearing trouble. They were also more likely to have a personal computer at home and use the internet, suggesting they might be good candidates for an online model of hearing care, such as through mobile health applications and tele-audiology. The coronavirus pandemic, which has significantly disrupted access to clinic-based health care, has increased the need for alternative models of hearing care, particularly those that incorporate an online or tele-audiology component. As evidenced in the UK during the pandemic, there remain both provider- and patient-side barriers to delivering tele-audiology services (37).

The purpose of this analysis is to better inform the planning for increasing access to hearing care at a time when the existing model is already undergoing substantial change. The introduction of the Over-the-Counter Hearing Aid Act (2017) which will regulate the sale of hearing aids to treat mild- to moderate-hearing loss over the counter and is expected to spawn a broader array of more affordable, quality-controlled devices available direct to the consumer. If these low-cost devices are placed in pharmacies, or doctors' offices, it may result in greater uptake of hearing care among older adults who have not previously engaged in the existing model of care.

With many alternative delivery models focused on increasing access to affordable devices without hearing care services come questions of quality of care. While early analysis of self-fitting devices compared to audiologist supported fitting suggests that comparable outcomes can be achieved (23), the literature suggests that hearing outcomes are optimized when receiving supportive hearing services in addition to the device (23, 38, 39). Further, greater perceived self-efficacy of managing hearing aids is associated with a more successful outcome in using and benefiting from a device (40). OTC hearing aids are designed to be self-fitting however, it is too early to tell whether device adherence and hearing outcomes under an OTC model will be maintained at least to the extent observed in hearing care service approaches. Certainly, device-focused approaches do not support or promote the development of coping strategies for psychosocial impacts of hearing loss (41), partly resulting from the chronic nature of hearing loss and the limitations of hearing aids to fully compensate for the impairment (42).

Ultimately, there is unlikely to be a one-size-fits-all approach to hearing care. Even across two common delivery models such as medical provider and prescription drug there was 34% overlap among Medicare beneficiaries with untreated functional hearing trouble. Alternative delivery methods should consider how to complement the existing model rather than substitute for it. Defining a pathway for hearing care in the U.S. that covers the spectrum from prevention of hearing loss to treatment, and addresses the physical, financial, and emotional barriers to seeking care is a crucial goal of the system. This will require engagement from older adults and current hearing care providers, as well as other stakeholders both inside and outside the health care system.

Many alternative or complementary models of care will require changes to existing workforce and reimbursement structures, including forward thinking on scope-of-practice legislation, investments in training and certification of paraprofessionals, such as community health workers, and the reimbursement of education and counseling separate from the cost of the device (18, 43).

The limitations of the study reflect the challenge of refining the measures for hearing loss and utilization based on the survey and administrative data available. Firstly, hearing loss is measured by self-reported trouble hearing, not by a professional examination. The population captured in this analysis is therefore those who recognize they have some degree of hearing trouble and does not reflect the entire population who could potentially benefit from some degree of hearing care. Previous studies have shown that individuals often underestimate their hearing loss and that underestimation can vary by sociodemographic characteristics (44). For the purposes of this analysis, however, we are interested in how to better reach those who recognize they have hearing difficulties, but are not using hearing aids.

Secondly, the categories of utilization provided by the MCBS contain broader categories of medical provider and prescription drugs than primary care and pharmacy visits, respectively. Our methods detail the ways that we attempted to refine these variables; however, it is possible that our estimates of potential reach of alternative delivery models may over-estimate those attending primary care clinics or pharmacies. The prescription drug measure does not account for visits to the pharmacy for over-the-counter purchases. It is therefore also possible that this pathway is under-estimated. Finally, we do not assume that attendance at these alternative pathways equates to receiving hearing care treatment.

This analysis highlights the ongoing inequities in the existing hearing care system which may be attributable to the financial and physical barriers associated with high-cost devices and clinic-based hearing care. Disruption to the clinic-based delivery model brought on by the introduction of FDA-regulated, direct-to-consumer hearing aids, has resulted in piloting of models in the community, pharmacies, and medical systems. This analysis suggests that these new delivery models may create greater equity in hearing care access by reaching currently unserved populations.

## Data Availability Statement

The data that supports the findings of this study are available from the Centers for Medicare and Medicaid Services (CMS) but restrictions apply to the availability of these data, which were used under license for the current study, and are not publicly available. Data can be requested from cms.gov.

## Author Contributions

AW, LA, and NR designed the study and drafted the manuscript. AW conducted the data analysis. AW, LA, CN, CM, FL, and NR interpreted the data. CN, CM, and FL critically revised the manuscript for important intellectual content. All authors contributed to the article and approved the submitted version.

## Funding

This work was supported by the Commonwealth Fund (grant number 20192345). This manuscript was also supported in part by funding from the Cochlear Center for Hearing and Public Health at the Johns Hopkins Bloomberg School of Public Health. CN was funded by NIA/NIH grant K23AG059900. NR was funded by NIA/NIH grant 1K23AG065443. Funders and sponsors had no role in the design, methods, analysis, or preparation of the paper.

## Conflict of Interest

AW reports receiving a speaker honorarium from the American Speech-Language-Hearing Association and consulting honorarium from BioMedical Insights. CN serves on the non-profit board of directors for Access HEARS and the Hearing Loss Association of America. NR is a scientific advisory board member of Shoebox, Inc and Good Machine Studio but does not receive financial compensation. FL reports being a consultant to Frequency Therapeutics, receiving speaker honoraria from Caption Call, and being the director of a public health research center funded in part by a philanthropic gift from Cochlear Ltd to the Johns Hopkins Bloomberg School of Public Health. CM is on the scientific advisory board for Good Machine Inc but does not receive financial compensation. The remaining author declares that the research was conducted in the absence of any commercial or financial relationships that could be construed as a potential conflict of interest.

## Publisher's Note

All claims expressed in this article are solely those of the authors and do not necessarily represent those of their affiliated organizations, or those of the publisher, the editors and the reviewers. Any product that may be evaluated in this article, or claim that may be made by its manufacturer, is not guaranteed or endorsed by the publisher.
