# Supplementary material for: Alternative Pathways for Hearing Care May Address Disparities in Access
Source: Front Digit Health. 2021 Nov 25;3:740323. doi: 10.3389/fdgth.2021.740323 (PMC8655113; doi:10.3389/fdgth.2021.740323)
Supplement: Supplementary file 1 [file Table_1.DOCX]

Supplementary Table S1: Description of Alternative Models of Hearing Care in Practice

| Alternative Models of Care | Description |
| --- | --- |
| Community | Community health worker models of care encapsulates a range of promising models increasingly incorporated into hearing care. Community health worker models can include community health workers, peer educators, community health aides, among other trained paraprofessionals who can provide education on hearing loss, basic aural rehabilitation, as well as fitting and orientation to OTC devices.(25, 26, 39) For example, under audiology supervision, older adult peer educators provide a 2-hour intervention, ‘HEARS’, that incorporates basics of age-related hearing loss, communication strategies, and step-by-step fitting and orientation to an OTC device through affordable senior housing and senior centers. |
| Telehealth | Traditionally telehealth audiology services has focused on screening and diagnostic measures to identify hearing loss via smartphones and portable systems. However, recent advances allow hearing aids to connect to smartphones via Bluetooth that create opportunity for remote adjustments of hearing aids or even automated fitting procedures. |
| Adult Day Clinics | Teams of researchers have applied the ‘HEARS’ model described above in dementia day clinics. Hearing was addressed in the PACE clinic by trained professionals and included significant training on use and upkeep of devices for all providers and staff involved in the clinic (e.g., physicians, nurses, cafeteria, transportation, etc.). |
| Retail Clinics | Currently, large retailers in the United States, such as Costco and Sam’s Club have integrated hearing aid centers into their stores. This model generally recreates best-practice hearing aid delivery models used in private clinics but increases accessibility by putting the clinic where customers/clients/patients already are shopping and increases affordability by leveraging buying power from the large corporations. |
| Pharmacies | CVS and Walgreens each have experimented with a similar model to the Retail Clinics; however, each eventually abandoned the traditional model in the pharmacies. Examples of pop-up hearing services in pharmacies exist in Australia and the United Kingdom whereby adult hearing checks are conducted with referral services available for those requiring further support. |
